# SOCIOECONOMIC STATUS, CHANGES IN HEALTH BEHAVIORS, AND DEPRESSIVE SYMPTOMS AMONG OLDER ADULTS DURING THE PANDEMIC

**DOI:** 10.1093/geroni/igad104.1875

**Published:** 2023-12-21

**Authors:** Yujun Liu, Heng Wang, M Courtney Hughes

**Affiliations:** Northern Illinois University, DeKalb, Illinois, United States; Rush University Medical Center, Chicago, Illinois, United States; Northern Illinois University, DeKalb, Illinois, United States

## Abstract

Disparities in COVID-19 morbidity and mortality based on race and gender exist among older adults. Few studies have explored the complex relationships between the multiple components of COVID-19 vulnerabilities and the intersectionality across gender and race. This study aims to examine the impact of the COVID-19 pandemic on financial difficulty, health behavior, and depressive symptoms among older adults across the intersectionality of gender and race. Using Round 5 and Round 10 of the National Health and Aging Trends Study (NHATS) linked with the NHATS COVID-19 dataset, our sample included Medicare beneficiaries aged 65 or older in the U.S. (N =3,118). We modeled the interrelationships between financial difficulty, health behaviors, and depressive symptoms using a structural equation model. The results indicate that within the intersectionality framework, female participants reported less walking, more changes in eating habits, less sleeping, and less alcohol consumption during the pandemic than before compared to male counterparts. Compared to Non-Hispanic White, Non-Hispanic Black, Hispanic, and Other races, participants showed higher proportions of experiencing financial difficulty, less walking, less vigorous activity and changes in eating and sleep during than before the pandemic. Financial difficulty was significantly and positively associated with depressive symptoms and sedentary behavior. Active behavior was significantly and negatively associated with depressive symptoms, sedentary behavior was significantly and positively associated with level of depressive symptoms. Health professionals should consider the connection between various sociodemographic and health factors and the impact of COVID-19 on older adults when designing and delivering health and wellness programs post-pandemic.

